# Treating firm, atypical striae with intralesional corticosteroids

**DOI:** 10.1016/j.jdin.2026.03.004

**Published:** 2026-03-19

**Authors:** Esther Nwozo, Osahon Iyamu, Michal Kidacki

**Affiliations:** a Lewis Katz School of Medicine at Temple University, Philadelphia, Philadelphia; b University of Pennsylvania Perelman School of Medicine, Philadelphia, Philadelphia; c Department of Dermatology, University of Iowa, Iowa City, Iowa

**Keywords:** collagen deposition, hypertrophic scarring, striae distensae, triamcinolone acetonide

## Challenge

Striae distensae are typically characterized by decreased collagen, reduced elastin, and epidermal atrophy. However, some variants may present as firm, hypopigmented plaques with increased dermal collagen, features that more closely resemble hypertrophic scarring than classic striae. These atypical presentations can create diagnostic uncertainty and complicate management, because most standard striae treatments (topical retinoids, collagen-stimulating lasers, and microneedling) are designed to increase collagen production, which may be counterproductive in collagen-rich variants.^1^ The challenge lies in identifying the collagen-rich striae and choosing the appropriate therapeutic approach.

**Fig 1:**
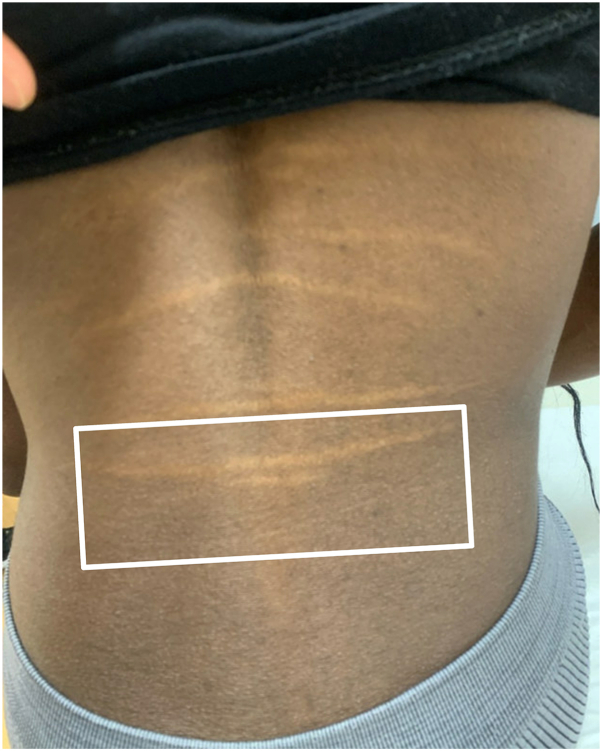
Linear, hypopigmented lesions prior to treatment initiation.

A 28-year-old woman presented with asymptomatic, hypopigmented linear plaques on her lower portion of the back, present since adolescence. Family history was positive for similar lesions in her father and 2 brothers. Examination revealed several horizontally oriented, hypopigmented-to-yellowish, palpable plaques across the lower portion of the back (Fig 1). Differential diagnosis included linear focal elastosis versus striae distensae. A punch biopsy obtained from the most inferior lesion demonstrated slightly decreased elastic tissue on Verhoeff-Van Gieson staining and collagen thickening on hematoxylin and eosin staining without clumped elastic fibers, favoring striae distensae.

The plaques’ firmness and scar-like appearance raised the question: how should clinicians approach striae when biopsy shows collagen excess rather than loss?

## Solution

**Fig 2:**
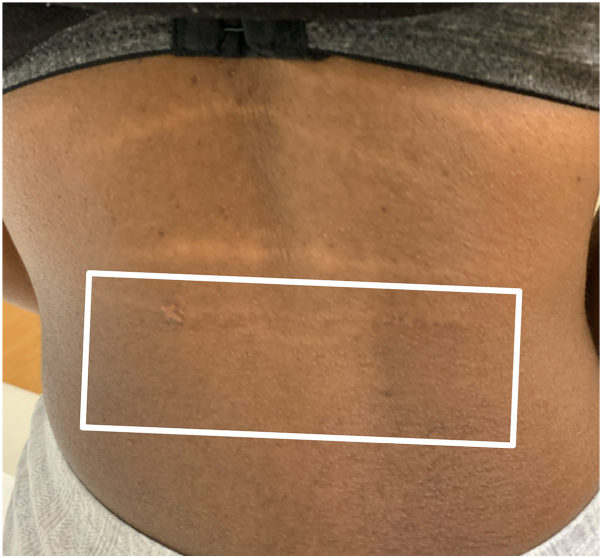
Lesions from most recent follow-up visit after 5 mg/mL triamcinolone acetonide injections. This image illustrates the softening and blending of lowermost lesion with surrounding skin. Biopsy scar visible on the left side.

The lowermost plaque was selected for initial treatment with liquid nitrogen cryotherapy followed by intralesional triamcinolone acetonide (TAC) 5 mg/mL to both frozen and unfrozen areas. At 3 months, the plaque became softer and began to repigment, with no difference between frozen and unfrozen regions. Afterward, the lowermost plaque was treated only with TAC 5mg/mL at months 3, 6, and 9, whereas the superior plaques were treated at months 6 and 9 only. At 12 months, the lowermost lesion demonstrated marked softening and blending with surrounding skin, whereas superior lesions demonstrated mild improvement. A plaque immediately above the lowermost lesion became more prominent on the left side, indicating ongoing disease (Fig 2). No adverse effects were reported.

Although corticosteroids can induce striae in classic, collagen-poor variants, thickened plaques detected by clinical examination and palpation may indicate underlying collagen deposition, supporting the use of TAC to reduce dermal fibrosis, consistent with the scar-like biology.^2^ Biopsy is not required in most cases, as physical examination is typically sufficient to guide management. Intralesional TAC may be an effective, underrecognized option for striae with increased collagen deposition and a firm, scar-like texture.

## Conflicts of interests

Dr Kidacki serves as a paid consultant for Guidepoint Global on topics unrelated to this work. All other authors declare no conflicts of interest.
